# Arbutin as a Skin Depigmenting Agent with Antimelanogenic and Antioxidant Properties

**DOI:** 10.3390/antiox10071129

**Published:** 2021-07-15

**Authors:** Yong Chool Boo

**Affiliations:** Department of Molecular Medicine, Cell and Matrix Research Institute, BK21 Plus KNU Biomedical Convergence Program, School of Medicine, Kyungpook National University, Daegu 41944, Korea; ycboo@knu.ac.kr; Tel.: +82-53-420-4946

**Keywords:** arbutin, melanin, pigment, melasma, skin lightening, cosmetic, hyperpigmentation, tyrosinase, antioxidant, nuclear factor erythroid 2-related factor 2 (Nrf2)

## Abstract

Arbutin is a compound of hydroquinone and D-glucose, and it has been over 30 years since there have been serious studies on the skin lightening action of this substance. In the meantime, there have been debates and validation studies about the mechanism of action of this substance as well as its skin lightening efficacy and safety. Several analogs or derivatives of arbutin have been developed and studied for their melanin synthesis inhibitory action. Formulations have been developed to improve the stability, transdermal delivery, and release of arbutin, and device usage to promote skin absorption has been developed. Substances that inhibit melanin synthesis synergistically with arbutin have been explored. The skin lightening efficacy of arbutin alone or in combination with other active ingredients has been clinically evaluated. Combined therapy with arbutin and laser could give enhanced depigmenting efficacy. The use of arbutin causes dermatitis rarely, and caution is recommended for the use of arbutin-containing products, especially from the viewpoint that hydroquinone may be generated during product use. Studies on the antioxidant properties of arbutin are emerging, and these antioxidant properties are proposed to contribute to the skin depigmenting action of arbutin. It is hoped that this review will help to understand the pros and cons of arbutin as a cosmetic ingredient, and will lead to future research directions for developing advanced skin lightening and protecting cosmetic products.

## 1. Introduction

**Figure 1:**
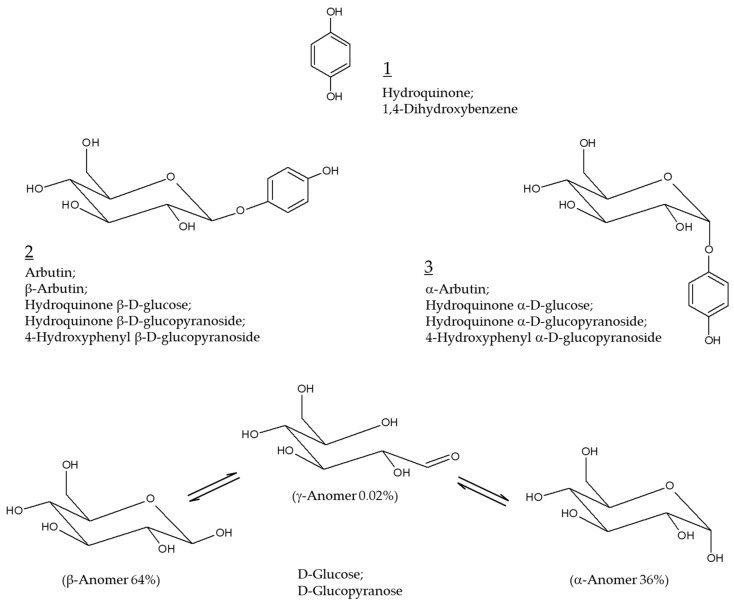
Chemical structures of hydroquinone, arbutin (β-arbutin), and α-arbutin. Anomeric structures of D-glucose are also shown for comparative purposes.

Hydroquinone (Chemical structure 1, Figure 1) at concentrations varying from 2 to 5% has been prescribed as the primary therapy for hyperpigmentation disorders including melasma [1,2]. It is used alone or in combination with other active pharmaceutical ingredients, such as retinoids and steroids, for added benefits [3,4]. Hydroquinone not only inhibits tyrosinase (TYR) activity and destroys melanosomes, but also causes necrosis of melanocytes by modifying the membrane structure [5]. This is the potential mechanism of action of hydroquinone as a skin lightening agent as well as its toxicity mechanism. The use of this ingredient in cosmetics has been banned since 2001 because of the high risk of carcinogenesis in case of prolonged exposure to hydroquinone [6,7].

Arbutin (Chemical structure 2, Figure 1) is a compound with a structure in which one molecule of D-glucose is bound to hydroquinone. D-glucose exists in α, β, or γ-anomeric form in aqueous solution, with β-anomer being a dominant form. β-Arbutin (this stereoisomer is called arbutin) in which the β-anomer of D-glucose is bound to hydroquinone is mainly found in plants, such as wheat, pear, and bearberry [8]. α-arbutin (Chemical structure 3, Figure 1) is a compound of hydroquinone and the α-anomer of D-glucose [9]. It has been about 30 years since arbutin was studied in earnest to use as a hydroquinone alternative for skin lightening purposes [10]. It is thus timely to investigate the accumulated information on the efficacy and safety of arbutin and its mechanism of action.

In this review, we will try to get answers to questions that have been controversial. The questions are as follows. Which arbutin or α-arbutin is more advantageous as a skin lightening agent? Do arbutin and α-arbutin have properties of inhibiting melanin synthesis without releasing hydroquinone? Is the toxicity of arbutin due to the release of hydroquinone?

There are other important questions to be answered. Is there a substance with a similar structure with more advantageous properties than arbutin? Could it be possible to increase the skin absorption efficiency of arbutin by a formulation or device-assisted method? Do clinical trial results support the skin lightening benefits of arbutin outweighs its potential harmful risks? What are the side effects of arbutin when applied to the skin? Do the antioxidant properties of arbutin and or α-arbutin contribute to their skin depigmenting action? Can arbutin be applied to skin disorders other than hyperpigmentation?

We hope that this review will help you find answers to these questions. It is also hoped that this review will help identify the pros and cons of arbutin as an active cosmetic ingredient, improve our understanding of its mechanism of action, and set the direction for future research on its cosmetic applications.

## 2. Modulation of Melanin Synthesis

### 2.1. Pigmentation and Melanin

Melanin is a polymeric, colored pigment distributed throughout the skin, hair, eye, and other tissues. It is synthesized in melanosomes, unique organelles located in epidermal melanocytes, and plays key roles in maintaining skin homeostasis [11,12], and photoprotection [13,14]. Dysregulated melanin metabolism results in skin pigmentary disorders, such as hyperpigmentation and hypopigmentation [15,16,17]. The melanocytes derived from individuals with different skin colors show different melanogenic activity although the distribution density of melanocytes in the skin is not much different [18,19]. There was a close association between the melanogenic activity of melanocytes and skin color in humans [20,21].

### 2.2. Regulation of Melanin Synthesis

Melanin synthesis is influenced by diverse factors including genetic background, epigenetic adaptation, hormonal changes, nutritional status, and environmental conditions [22,23]. For example, proopiomelanocortin-derived peptide hormones, such as α-melanocyte-stimulating hormone (MSH), β-MSH, and adrenocorticotrophic hormone, act as agonists of the melanocortin 1 receptor, a G protein-coupled receptor, and stimulate protein kinase A-mediated signaling pathway leading to the activation of cAMP response element-binding protein (CREB). [22,24]. In the nucleus, the active CREB binds to the cAMP response element on the promoter of microphthalmia-associated transcription factor (MITF), inducing transcription of the target gene [25,26]. MITF plays a key role in the regulation of melanin synthesis by regulating gene expression of melanogenic enzymes, including TYR, tyrosinase-related protein-1 (TYRP-1), and tyrosinase-related protein-2 (TYRP-2) [22,24]. MITF can also be activated by the stem cell factor/receptor tyrosine kinase protein c-Kit/mitogen-activated protein kinases *pathway,* and Wnt/frizzled/glycogen synthase kinase 3β/β-catenin pathway [27,28]. Other signaling pathways, such as phospholipase C/diacylglycerol/protein kinase Cβ cascade, and nitric oxide/cGMP/protein kinase G cascade also can regulate melanin synthesis [29,30]. For more details, please refer to other reviews on autocrine and paracrine regulation and cell signaling pathways associated with melanogenesis [30,31].

### 2.3. Melanin Synthesis Pathway

Melanin synthesis begins with the oxidation of L-tyrosine to DOPAquinone by monophenolase activity of TYR or the oxidation of L-3,4-dihydroxyphenylalanine (DOPA) to DOPAquinone by diphenolase activity of TYR [32,33,34,35]. DOPAquinone reacts with cysteine to produce 5-S-cysteinylDOPA or 2-S-cysteinylDOPA and enters the pheomelanin synthesis pathway. These two compounds are oxidized to quinones and through intramolecular cyclization, and benzothiazine and benzothiazole intermediates are produced. These intermediates are used as building blocks to synthesize the reddish-yellow polymer pheomelanin [36]. Alternatively, when DOPAquinone is oxidized to DOPAchrome via leukoDOPAchrome, it enters the eumelanin synthesis pathway. DOPAchrome is converted to 5,6-dihydroxyindole-2-carboxylic acid (DHICA) by DOPAchrome tautomerase activity of TYRP-2 or to 5,6-dihydroxyindole (DHI) by releasing CO_2_. DHICA and DHI are oxidized to quinones by the DHICA oxidase activity of TYRP-1, and the DHI oxidase activity of TYR, respectively, and these are used as building blocks to synthesize the brownish-black polymer eumelanin [37].

### 2.4. Artificial Modulation of Melanogenesis

TYR-catalyzed enzyme reactions constitute key steps in the biosynthetic routes for melanin, and thus, the enzyme provides a useful target for the pharmacological control of skin hyper- and hypo-pigmentation in dermatology and cosmetology [38,39]. Gene expression of TYR could be enhanced or suppressed via pharmacological approaches [14,40]. Various synthetic and natural compounds are known to inhibit the catalytic activity of TYR in vitro [41,42,43]. For some active ingredients, the skin depigmenting efficacy was verified through clinical trials [44,45,46,47,48]. Various depigmenting active ingredients including arbutin are used in cosmetics [49].

## 3. Arbutin

### 3.1. Anti-Melanogenic Effect of Arbutin

Arbutin has the effect of reducing melanin content at a concentration that has little effect on the viability of cultured human melanocytes. Maeda et al. showed that arbutin dose-dependently reduced TYR activity in human melanocytes at concentrations between 0.1 and 1.0 mM without significantly decreasing cell viability, and its inhibitory effect against cellular melanin synthesis was more potent than that of kojic acid or L-ascorbic acid when compared at a fixed concentration (0.5 mM) [50]. Akiu et al. reported that the melanin content of cultured murine melanoma B16 cells was reduced by arbutin, and the effect was explained by the decrease in intracellular TYR activity [10]. Arbutin was shown to inhibit melanin production in B16 cells stimulated by α-MSH and abrogate the hyperpigmentation effects of α-MSH in brownish guinea pig and human skin explants in organ culture experiments [51].

The decrease in TYR activity in human melanocytes by arbutin does not appear to be due to the decrease in the expression level of this enzyme. Maeda et al. reported that arbutin at 0.5 mM reduced the activity of intracellular TYR by 50% but did not affect the mRNA expression level of TYR [52]. Chakraborty et al. showed that arbutin (0.37 mM) lowered cellular melanin content but did not reduce the protein levels of TYR, TYRP-1, and TYRP-2 [53]. If so, arbutin may inhibit post-translational modification or maturation of newly synthesized TYR or may induce irreversible inactivation of already synthesized mature TYR. In an experiment in which lysates obtained from human melanocytes treated with 1.0 mM arbutin or not were analyzed by zymography, the TYR activity was reduced to 87% in the former [52]. Therefore, it is reasonable to consider arbutin as an inactivator of cellular TYR, rather than a suppressor of TYR gene expression.

In an in vitro assay using crude protein extracts derived from murine melanoma B16 cells, arbutin was shown to be able to directly inhibit the catalytic activity of TYR [10]. In the experiment with mushroom TYR, when L-DOPA was used as a substrate, arbutin showed a lower inhibitory effect than kojic acid and L-ascorbic acid: Their 50% inhibitory (IC_50_) values were 10 mM, 0.12 mM, and 0.2 mM, respectively [52]. In the experiment using human TYR (derived from melanocytes of Asian neonatal foreskins), when either L-tyrosine (for monophenolase activity) or L-DOPA (for diphenolase activity) were used as substrates, the IC_50_ values of arbutin were 5.7 mM and 18.9 mM, respectively: Arbutin appeared to be an inhibitor in a competitive relationship with L-tyrosine [52]. It is presumed that arbutin competes with a structurally similar substrate to bind to the active site of the TYR enzyme. More importantly, the concentration at which arbutin inhibits the catalytic activity of TYR in vitro is higher than the concentration that reduces cellular melanin, so there is no conviction as to whether this mechanism works in the cell.

**Figure 2:**
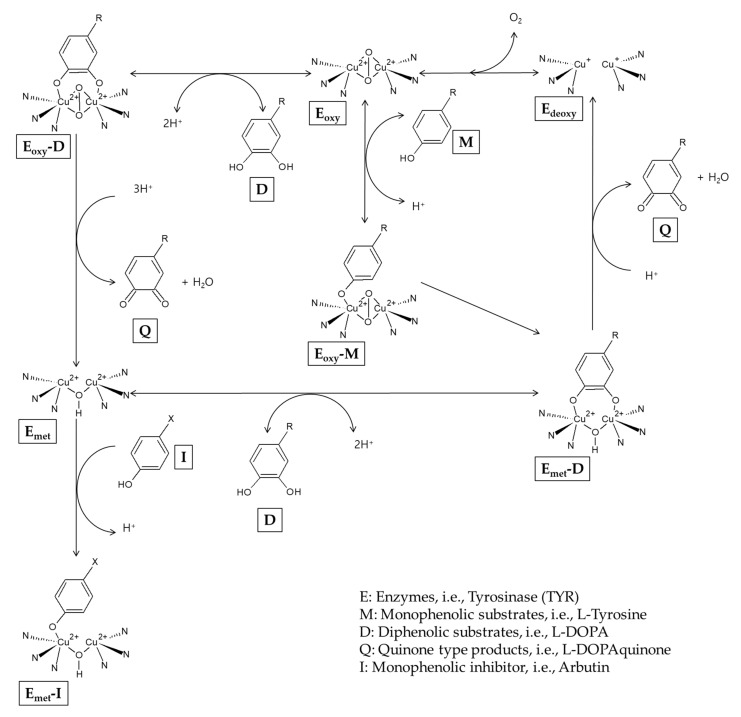
A potential mechanism for the inactivation of tyrosinase by arbutin.

It has been reported that arbutin can also act as a substrate for TYR. In the presence of a catalytic amount of L-DOPA as a cofactor, arbutin is oxidized by mushroom TYR to produce 3,4-dihydroxyphenyl-O-beta-D-glucopyranoside [54]. During the catalysis, the TYR enzyme exists as E_met_, E_deoxy_, and E_oxy_ forms that are mutually converted [34] (Figure 2). E_deoxy_ binds oxygen to form E_oxy_ that can capture a monophenol (M) or diphenol (D) substrate. When E_oxy_ encounters an M substrate, E_oxy_-M is formed and it is converted to E_met_-D that releases a quinone (Q) product and regenerates E_deoxy,_ completing the monophenolase cycle. If E_oxy_ encounters a D substrate, E_oxy_-D is formed and then releases a Q product and E_met_. E_met_ can bind to another D substrate, forming E_met_-D that releases a Q product and E_deoxy,_ completing the monophenolase cycle. E_deoxy_ proceeds to the next cycles. However, in the absence of D substrates, E_met_ binds to a monophenol inhibitor (I), and this results in a dead-end pathway, causing the inactivation of the enzyme. Arbutin is considered to be able to inactivate the TYR enzyme by binding with E_met_ under L-DOPA-deficient conditions [55].

Inoue et al. compared the effects of hydroquinone and arbutin on the differentiation of melanocytes [56]. The results showed that hydroquinone downregulated the early stage of differentiation of mouse embryonic stem cells to neural crest cells, and the late stage of differentiation to melanocytes with melanogenic capability. On the other hand, arbutin did not affect the early and late stages of differentiation of melanocytes and only suppressed elevations in TYR expression in the late stage of differentiation.

### 3.2. A Possible Production of Hydroquinone from Arbutin

There is a disagreement over whether arbutin works by being decomposed into hydroquinone and glucose or not. Akiu et al. stated that the decomposition of arbutin in the cell suspension could not be observed [10]. However, as shown in the experiment of Maeda et al., hydroquinone can reduce the activity of intracellular TYR by 50% at a concentration 100 times lower than arbutin [50,52]. When arbutin is added to cosmetic products, hydroquinone can be produced to a different level depending on storage conditions [57,58]. In addition, when arbutin is applied to the skin, hydroquinone can be produced by exposure to skin microorganisms [59] or ultraviolet radiation (UVR) [60]. Therefore, there remains a possibility that a small amount of hydroquinone, which may be produced as a decomposition product of arbutin, contributes to the inhibition of melanin synthesis or the inactivation of TYR in cells. Nevertheless, the majority of evidence supports that arbutin has intrinsic properties that inhibit cellular melanogenesis and reduce cellular TYR activity regardless of hydroquinone release.

### 3.3. Pro-Melanogenic Effect of Arbutin

There is another report that conflicts with many other studies that have reported the melanin-lowering action of arbutin. Nakajima et al. observed that in cultured normal human melanocytes (from neonatal Caucasian foreskins) treated with increasing concentrations of arbutin, the pigmentation became darker (effective concentrations, 2–8 mM), whereas the viability (2–8 mM) and the TYR activity (0.5–4 mM) of the cells decreased [61]. One possible explanation for the particular observation is that arbutin increases the synthesis of intracellular melanin by acting as a substrate of TYR as discussed above. Another possibility is that although arbutin inhibits the synthesis of new melanin, it blocks the release of synthesized melanin out of the cell, thereby causing the accumulation of melanin inside cells. Melanosome transfer is a unique biological process that delivers a package of organelles from melanocytes to keratinocytes [62], and various mechanisms, such as cytophagocytosis, membrane fusion, shedding-phagocytosis, and exocytosis-endocytosis, have been proposed to explain this process [63].

### 3.4. Preparation of Arbutin

Arbutin can be prepared by various methods, such as extraction from plants, bioconversion from hydroquinone, and chemical synthesis [64,65]. The content of arbutin in a plant varies depending on the species, parts of the plant, development stages, and harvest season [66,67]. The production efficiency for arbutin also varies depending on the extraction and purification methods [68,69,70].

Fermentation of soybeans with certain strains of Bacillus subtilis was shown to produce arbutin and other TYR inhibitory compounds [71]. Shang et al., engineered a yeast, Yarrowia lipolytica, to produce arbutin by expressing three exogenous genes, such as chorismate pyruvate lyase, 4-hydroxybenzoate 1-hydroxylase, and hydroquinone glucosyltransferase [72]. The engineered Y. lipolytica was capable of de novo biosynthesis of arbutin through the shikimate pathway.

## 4. α-Arbutin

### 4.1. Preparation of α-Arbutin

α-arbutin could be produced through transglycosylation from various glucose donors to hydroquinone catalyzed by purified enzymes. Sucrose phosphorylase from Leuconostoc mesenteroides [73], α-amylase from Bacillus subtilis X-23 [74], sucrose isomerase from Erwinia rhapontici [75], amylosucrase from Deinococcus geothermalis [76] and Cellulomonas carboniz T26 [77], and cyclodextrin glucanotransferase from *Thermoanaerobacter* sp. [78] were capable of α-arbutin synthesis.

Kurosu et al. synthesized α-arbutin through fermentation of Xanthomonas campestris WU-9701 [79], Xanthomonas CGMCC, and Xanthomonas BT-112 [80,81]. Wu et al. developed a fed-batch culture strategy for the production of α-arbutin using recombinant Escherichia coli cells anchoring surface-displayed transglucosidase as a whole-cell biocatalyst and lactose as an inducer of the recombinant protein [82]. α-arbutin can be chemically synthesized via an approach similar to that for arbutin [83,84].

### 4.2. Anti-Melanogenic Effect of α-Arbutin

Many studies have compared the inhibitory effects of arbutin (β-arbutin) and α-arbutin on TYR catalytic activity in an in vitro experiment, with inconsistent results (Table 1). Kiato et al. reported that α-arbutin inhibited monophenolase activity of mushroom TYR with potency slightly lower than arbutin or hydroquinone [73]. Funayama et al. reported that α-arbutin inhibited diphenolase activity of TYR derived from murine melanoma 10 times more potently than β-arbutin, and their IC_50_ values were 0.48 mM and 4.8 mM, respectively [85]. On the other hand, the inhibitory effect against mushroom TYR diphenolase was not observed in the case of α-arbutin, unlike β-arbutin (IC_50_, 8.4 mM). Later, Qin et al. reported that α-arbutin inhibited monophenolase activity (IC_50_, 4.5 mM), whereas it activated diphenolase activity of mushroom TYR [86].

Garcia-Jimenez et al. demonstrated that both α and β-arbutin are apparent competitive inhibitors against both the monophenolase and diphenolase activities of TYR [87]. In their study, IC_50_ values of β-arbutin for TYR monophenolase and diphenolase activities were 0.9 and 0.7 mM, respectively, which were much lower than those for α-arbutin (IC_50_, 8.0 and 8.87 mM for TYR monophenolase and diphenolase activities, respectively). They also kinetically characterized α and β-arbutin as substrates of TYR and obtained their Michaelis constant values of 6.5 mM and 3.0 mM, respectively, supporting that the TYR enzyme has a higher affinity for β-arbutin than α-arbutin.

As such, contradictory results have been reported regarding which arbutin or α-arbutin is a more potent TYR inhibitor. The causes for this extreme inconsistency between studies are not clear and it is only assumed that the inconsistent results may be due to differences in the origin and purity of the enzyme, the conformational state of the enzyme, the type and concentration of substrates, oxygen concentration, pH, temperature, the purity of arbutin and α-arbutin, and the possibility of hydroquinone contamination or production. A conclusion could be reached if several institutions conduct studies comparing the activity of the same substances under standardized experimental conditions.

The antimelanogenic effects of α-arbutin were reported by Sugimoto et al. [88]. They showed that α-arbutin decreased melanin content and TYR activity in cultured human melanoma cells, at a concentration below 1.0 mM, without significant effects on cell growth and TYR mRNA level. They further showed that treatment of the human skin model with α-arbutin (250 μg per tissue) reduced melanin content to a 40% level of the control, without causing cell death.

## 5. Other Related Compounds

### 5.1. Glycosidic Derivatives of Arbutin and α-Arbutin

**Figure 3:**
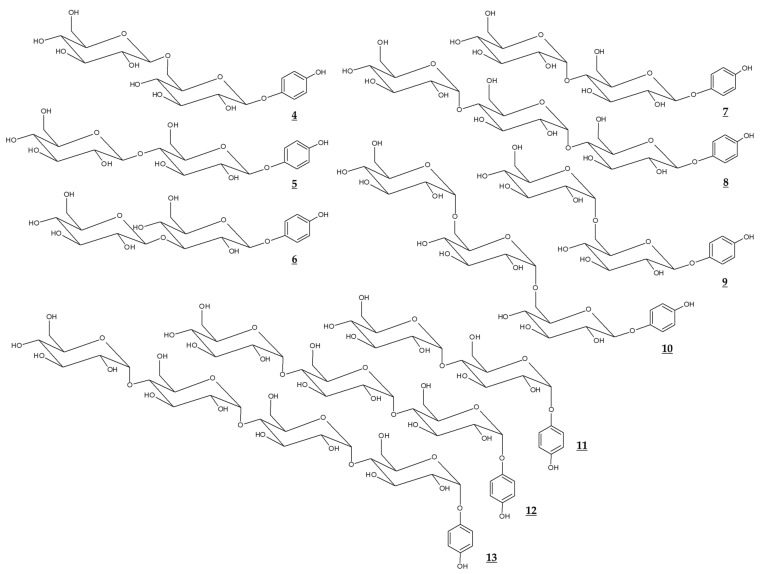
Chemical structures of glucosides of arbutin and α-arbutin.

Various glycosidic derivatives of arbutin and α-arbutin, which have additional molecules of glucose, were synthesized through a biological process (Table 2, Figure 3). It is interesting to note that their TYR inhibitory effects are different depending on the structure. However, they have a disadvantage in that the molecular weight is too large for industrial use.

### 5.2. Esters of Arbutin

**Figure 4:**
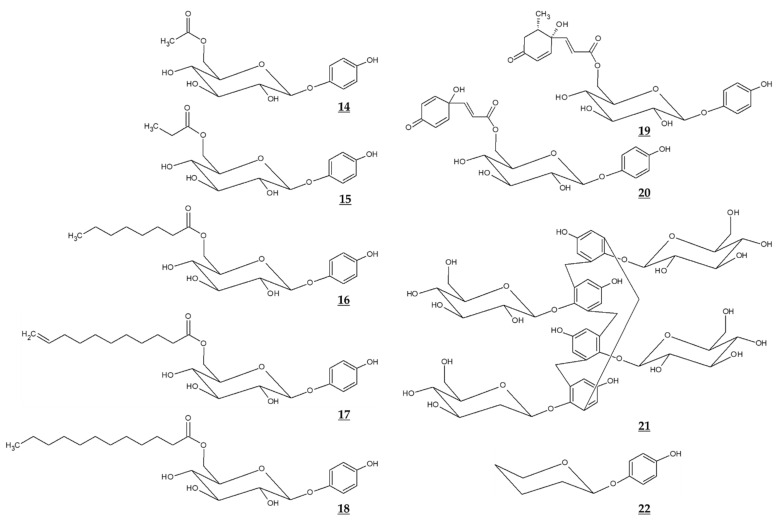
Chemical structures of various derivatives of arbutin and related compounds.

Jiang et al. compared the effects of arbutin and acetylated arbutin (Chemical structure 14, Figure 4) on cell viability, apoptosis, and migration as well as melanin synthesis capacity [94]. After a 24 h treatment at 5.4 mM, both arbutin and acetylated arbutin reduced cell viability and melanogenic capacity, induced cell apoptosis, G1 cell cycle arrest, and mitochondrial disruption in B16 murine melanoma cells, with the latter compound being more effective.

Various ester compounds of arbutin were also synthesized or extracted from plants, and some of them had TYR inhibitory effects or cell melanin synthesis inhibitory effects (Table 3, Figure 4). In particular, arbutin undecylenic acid ester (Chemical structure 17, Figure 4) was shown to be a more potent inhibitor of TYR than arbutin in several studies [95,96,97]. It is hoped to evaluate its skin lightening efficacy through in vivo studies or clinical trials.

Yamashita-Higuchi et al. isolated arbutin derivatives and related compounds from the leaves of Grevillea robusta [98]. Several compounds, such as grevilloside M and robustaside, showed potent antimelanogenic effects in B16 cells. These compounds were considered to inhibit melanin synthesis without involving TYR inhibition.

### 5.3. Calixarbutin

Ghaffarzadeh et al. synthesized calixarbutin (Chemical structure 21, Figure 4), a cyclic tetramer of arbutin that inhibits mushroom TYR activity and proliferation of A375 human malignant melanoma cell line, more potently than arbutin does [99]. The effects of calixarbutin on cellular melanogenesis and skin pigmentation are not known. Nonetheless, its cytotoxicity is very strong, and the molecular weight is too big to be used as an anti-melanogenic agent for topical application. Rather, its use for anticancer purposes is expected.

### 5.4. Deoxyarbutin

Deoxyarbutin (Chemical structure 22, Figure 4) was shown to be an effective inhibitor of mushroom TYR in vitro more potent than hydroquinone and arbutin [100,101]. In a hairless, pigmented guinea pig model, deoxyarbutin demonstrated rapid and sustained skin lightening whereas hydroquinone induced a transient skin lightening effect, and kojic acid and arbutin exhibited no significant skin lightening effect [100]. In a human clinical trial, topical treatment of 3% deoxyarbutin for 12 weeks improved solar lentigines in dark skin individuals, although it slightly reduced skin lightness in a population of light skin [100].

Another double-blind randomized controlled study was conducted treating a total of 59 women participants with 2% deoxyarbutin or 4% hydroquinone serum for 12 weeks [102]. During this period, the melanin index changed from 246.88 to 199.61 in the deoxyarbutin-treated group and from 244.22 to 203.98 in the hydroquinone-treated group, and skin lightness (L value) changed from 52.41 to 54.08 in the deoxyarbutin-treated group, and from 52.58 to 54.08 in the hydroquinone-treated group. Thus, it was concluded that that 2% deoxyarbutin and 4% hydroquinone sera showed comparable depigmenting efficacy. Despite these positive clinical results, there is a concern that deoxyarbutin may be degraded to produce hydroquinone, which may cause toxicity (Table 4).

## 6. Formulation and Devise

Arbutin was shown to be stabilized in lipid aggregates [106]. Monomyristoylphosphatidylcholine (14:0 lysoPC) and arbutin formed vesicles with interdigitated bilayers, stabilized by hydrogen bonds between glucose moiety of arbutin and the hydrated population of the carbonyl groups and the phosphates of lysoPC, and hydrophobic interactions between the phenyl group of arbutin and the acyl chain of lysoPC. The inclusion complex of arbutin in hydroxypropyl-β-cyclodextrin increased the water solubility and heat stability of arbutin [107].

**Figure 5:**
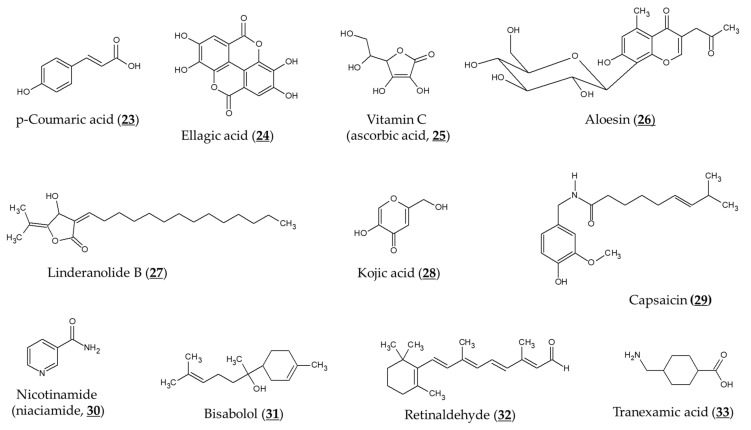
Chemical structures of various compounds that can affect the depigmenting activity of arbutin. Most compounds can additively or synergistically enhance the activity of arbutin, whereas capsaicin antagonizes the activity of arbutin.

Various approaches were developed to improve transdermal delivery and release of arbutin and α-arbutin. Encapsulation of arbutin in liposomes or micelles can enhance transdermal delivery and skin deposition [108,109]. Huang et al. designed nano-sized multi-phase emulsion using hydrocolloids for co-delivery of hydrophilic arbutin and hydrophobic p-coumaric acid (Chemical structure 23, Figure 5) [110]. The multi-phase emulsion showed a controlled release of both active ingredients. Chitosan nanoparticles and gold nanoparticles containing α-arbutin or β-arbutin have been prepared to enhance its transdermal delivery and release [111,112].

Liao et al. reported that transdermal delivery of α-arbutin could be enhanced by ultrasound-assisted technology [113]. Ultrasound treatment using albumin-shelled microbubbles as a contrast agent improved in vitro penetration of α-arbutin through C57BL/6J mouse skin. The microbubble/ultrasound-assisted treatment of α-arbutin solution for 4 weeks enhanced skin lightness of mice, more effectively than conventional treatment of α-arbutin solution. Aung et al. introduced α-arbutin-loaded dissolving microneedles and hydrogel-forming microneedles [114,115]. The microneedles showed enhanced intradermal delivery and skin accumulations of α-arbutin in vivo in mice compared to commercial α-arbutin cream. Kim et al. constructed arbutin-imprinted biomaterials for use in facial mask products [116].

## 7. Clinical Evaluation of Skin Depigmenting Efficacy

### 7.1. Skin Depigmenting Efficacy of Arbutin

Choi et al. evaluated the depigmenting efficacy of aloesin and arbutin in a human study [117]. After irradiating the skin area of the forearm with UVR, a 10% solution of each substance was treated alone or together 4 times daily for 15 days. As a result, aleosin, arbutin, and their co-treatment reduced the UVR-induced hyperpigmentation by 34%, 43.5%, and 63.3%, respectively compared to the vehicle treatment.

A randomized, prospective, open-label study of female patients aged 26–50 years with epidermal or mixed melasma evaluated the skin-lightening effects of arbutin and ellagic acid (Chemical structure 24, Figure 5) [118]. A gel formulation containing arbutin (1%), ellagic acid (1%), or ellagic acid plus plant extract (each 1%) was applied to the face twice a day for 6 months, and the skin melanin index was measured before and after using the product. The above three gel formulations reduced the melanin index to 71% (*p* = 0.05), 79% (*p* = 0.38) and 76% (*p* < 0.05) levels of baseline, respectively. No evaluation of control products without an active ingredient was performed and this is a limitation of this study.

A randomized, placebo-controlled, double-blind trial involving 102 women, aged 26– 55, with melasma and solar lentigines, evaluated depigmenting efficacy of arbutin derived from Serratulae quinquefoliae [119]. The study group (*n* = 54) applied the cream containing the plant extract (final concentration of arbutin 2.51%) twice a day on the discolored side for 8 weeks. The results showed that the cream with the plant extract decreased melanin level in the skin pigmentation spot, compared to the control group (*n* = 48) applied with a placebo cream without the active ingredient. During 8 weeks of application, the melanin level of the test group decreased from 182.60 ± 39.41 to 168.76 ± 36.30 (*p* < 0.000001), and there was no significant change from 158.9 ± 34.41 to 166.84 ± 39.72 in the control group. Clinical improvement was observed in 75.86% of the female patients with melasma and 56.00% of the female patients with solar lentigines.

### 7.2. Combination with Other Active Ingredients

TYR inhibitory activity of arbutin was shown to be enhanced by combination with L-ascorbic acid (Chemical structure 25, Figure 5), particularly when oxygen is limited [55]. Co-treatment of aloesin (Chemical structure 26, Figure 5) and arbutin was shown to inhibit mushroom TYR activity synergistically [120]. These two compounds also reduced the TYR activity and melanogenesis of cultured human melanocytes synergistically [121]. Synergistic effects inhibiting TYR activity and reducing melanin content of B16F10 cells were also observed between linderanolide B (Chemical structure 27, Figure 5), a natural compound purified from Cinnamomum subavenium, and arbutin or kojic acid (Chemical structure 28, Figure 5) [122]. The inhibitory effect of arbutin on TYR expression and melanin synthesis was reversed by capsaicin (Chemical structure 29, Figure 5) in B16 mouse melanoma cells [123].

There have been several studies evaluating a combination formulation containing arbutin in clinical trials. In a prospective, single-arm, open-label study involving 33 participants with epidermal melasma [124], a cream formulation containing 4% nicotinamide (niacinamide, Chemical structure 30, Figure 5), 3% arbutin, 1% bisabolol (Chemical structure 31, Figure 5), and 0.05% retinaldehyde (Chemical structure 32, Figure 5) was applied to the entire face once daily for 60 days. There was a mean reduction in MASI (melasma area and severity index) score for 60 days (2.25 ± 1.87, *p* < 0.0001). The mean total melasma surface was significantly reduced from 1398.5 mm^2^ at baseline to 923.4 mm^2^ at day 60 (*p* < 0.0001).

In a three-arm randomized controlled trial involving a total of 44 subjects [125], treatment of a combination serum that contained 4% ferment filtrate, 2% niacinamide, 4% α-arbutin, and 2% or 3% tranexamic acid (Chemical structure 33, Figure 5) showed a significant improvement in skin brightness and pigmentation intensity after 4 weeks (*p* < 0 .001). There were no differences in skin depigmenting efficacy between the combination serum treatment groups and the 4% hydroquinone treatment group.

A prospective, randomized, controlled split-face study [126] evaluated the hyperpigmentation improvement effect of a non-hydroquinone topical formulation (SKNB19) containing epidermal growth factor, tranexamic acid, vitamin C, arbutin, niacinamide, and other ingredients and a standard formulation containing 4% hydroquinone. Eighteen adult subjects with facial pigmentation were randomly assigned to apply SKNB19 twice daily to one side of their face and 4% hydroquinone at night to the other side. SKNB19-treated skin showed a statistically significant improvement in the overall appearance of hyperpigmentation and was rated better than hydroquinone-treated skin.

### 7.3. Combination Therapy with a Laser Treatment

A prospective study of 35 refractory melasma cases treated with 10 weekly laser sessions using a Q-switched Nd:YAG laser (MedLite C6), two monthly follow-up laser sessions, and topical 7% α-arbutin solution supported that the combination therapy is an effective and well-tolerated treatment for refractory melisma [127]. At 6 months, 66.7% of study subjects showed more than a 51% reduction of melisma even though mild and transient side effects were observed in several cases.

## 8. Antioxidant Properties of Arbutin and α-Arbutin

### 8.1. Reactive Oxygen Species in Melanin Synthesis

Melanin plays an important role in skin protection by absorbing UVR [128], but conversely, its synthetic process including reactions catalyzed by TYR generates reactive oxygen species (ROS) [129]. Independently of this, UVR, pollution, hormones, and drugs can stimulate the generation of ROS in melanocytes [130,131,132,133].

ROS of various origins can promote melanogenesis or cause melanocyte death, leading to hyperpigmentation or hypopigmentation [134]. Accumulation of oxidative damage leads to tumorigenesis [135]. Therefore, effective antioxidants are expected to reduce oxidative stress in cells, normalize the process of melanin production, and prevent melanocyte death and tumorigenesis. Oral administration of glutathione attenuated oxidative stress as well as hyperpigmentation [136,137].

### 8.2. Nuclear Factor Erythroid 2-Related Factor 2 (Nrf2)-Mediated Pathway

Cells have various forms of antioxidant defense, including the Nrf2-mediated pathway [40,138]. Nrf2 is a transcription factor that binds to antioxidant responsive elements (ARE) on the promoter regions of many phase II metabolism/antioxidant enzymes [139]. Activation of the Nrf2-ARE pathway leads to induction of phase II metabolic/antioxidant enzymes and increase of the low molecular antioxidants, such as glutathione [140].

Nrf2 was shown to negatively regulate melanin synthesis in cells through modulation of phosphoinositide 3-kinase/protein kinase B signaling pathway [141]. Dietary phenolics could suppress UVR-induced melanogenesis through stimulation of the Nrf2-ARE pathway in addition to their antioxidant and UVR absorption properties [142].

### 8.3. Antioxidant Properties of Arbutin and α-Arbutin

Takebayashi et al. examined the antioxidant activity of arbutin compared to that of hydroquinone [143]. Arbutin was a weaker scavenger against 1,1-diphenyl-2-picrylhydrazyl radical and a more potent scavenger against 2,2′-azinobis (3-ethylbenzothiazoline-6-sulphonic acid) cation radical compared to hydroquinone. Arbutin exerted slower but long-lasting scavenging activity against 2,2′-azobis (2-methylpropionamidine) dihydrochloride (AAPH)-derived peroxyl radical compared to hydroquinone. At 50 μM arbutin prevented AAPH-induced hemolysis of erythrocytes more effectively than hydroquinone. Arbutin (125–1000 μM) rescued skin fibroblasts exposed to AAPH, whereas hydroquinone (125 μM) exerted cytotoxicity.

Tada et al. detected hydroxyl radical generation from the TYR-catalyzed oxidations of L-tyrosine and L-DOPA, using an electron spin resonance-spin trapping technique [144]. They also observed that arbutin attenuated the hydroxyl radical generation in both reactions, suggesting that arbutin can reduce the levels of ROS derived from the melanogenic pathway. Arbutin at 500 μM was also shown to reduce intracellular hydroxyl radical production and prevent mitochondrial membrane potential loss and played an anti-apoptotic role in human lymphoma U937 cells irradiated with X-ray [145]. Arbutin attenuated the tert-butyl hydroperoxide-induced oxidative stress in human liver cancer HepG2 cell line (effective concentration, 150 μM) [146], human prostate cancer LNCaP cell line, and human fibroblasts (250 and 1000 µM) [147].

Polouliakh et al. conducted in silico comparative genomics analysis in human dermal fibroblasts treated with α-arbutin, and identified transcription factors with a potential role in tumor suppression, toxicity response, and wound healing [148]. α-arbutin upregulated Nrf2 transcription factor which consequently activates target genes involved in antioxidant defense. Arbutin attenuated lipopolysaccharide-induced acute kidney injury in rats, by inhibiting inflammation and apoptosis via the phosphoinositide 3-kinase/protein kinase B/Nrf2 pathway [149]. Arbutin also decreased the levels of pro-inflammatory cytokines and enhanced myocardial antioxidant status, attenuating isoproterenol-induced cardiac hypertrophy in mice [150].

Thus, arbutin and α-arbutin may reduce ROS levels by directly scavenging free radicals or indirectly enhancing the antioxidant capacity of cells through the activation of the Nrf2-ARE pathway. These antioxidant properties may contribute to the inhibitory action of arbutin and α-arbutin on melanin synthesis in cells.

## 9. Discussion

The generally agreed view on the mechanism by which arbutin inhibits the melanin synthesis in cells is that it inhibits the catalytic activity of already expressed TYR or irreversibly inactivates it rather than suppressing the new synthesis of TYR. Many studies reported that arbutin did not affect mRNA and protein expression of TYR in the concentration range where it inhibited cellular melanin synthesis. There are only a few reports that arbutin reduced the protein level of intracellular TYR [123].

Arbutin is structurally similar to L-tyrosine and can bind to the active site of TYR, thereby acting as a competitive inhibitor. In addition, arbutin can irreversibly inactivate TYR by binding to the enzyme in the form of E_met_ when diphenol substrates are deficient. Of course, there is a possibility that arbutin may act as a substrate for TYR in the form of E_oxy_ when diphenol substrates are present. The last mechanism may not be excluded, because arbutin has been shown to increase cellular melanin synthesis at certain conditions [61]. However, most studies support the mechanism by which arbutin inhibits the catalytic activity of TYR or causes its irreversible inactivation, thereby preventing the synthesis of melanin in cells.

Arbutin exhibits different levels of toxicity depending on the cell type and exposure time, but 1 mM is considered as a boundary concentration between cytotoxicity and safety (Table 5). Therefore, the alleviating action of arbutin on cellular melanin synthesis and oxidative stress observed at a concentration of 1 mM or lower is interpreted as physiologically significant. When arbutin is treated in vivo, its concentration in contact with cells must be maintained at 1 mM or lower, so that beneficial efficacy without the risk of serious side effects can be expected.

**Figure 6:**
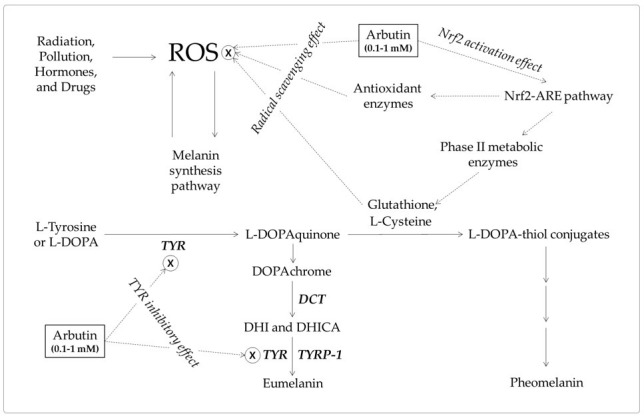
A hypothetical mechanism for the inhibition of eumelanin synthesis by arbutin involving its tyrosinase (TYR) inhibitory and antioxidant activities. Arbutin inhibits the catalytic activity of TYR. It also scavenges reactive oxygen species (ROS) from various sources that can induce melanin synthesis, apoptosis, or tumorigenesis. It can activate the erythroid 2-associated factor 2 (Nrf2)-antioxidant responsive elements (ARE) pathway to enhance the antioxidant capacity of cells. Increased thiol compounds, such as L-cysteine and glutathione, can react with L-DOPAquinon to form DOPA-thiol conjugates, which enter the pheomelanin synthesis pathway. As a result, eumelanin synthesis via DOPAchrome can be selectively downregulated by arbutin. Inhibitory targets of arbutin are indicated with ⊗.

Studies on the antioxidant activity of arbutin are emerging [143,147]. Arbutin scavenges ROS, such as hydroxyl radicals [144], and activates the Nrf2-ARE pathway enhancing the antioxidant capacity of cells [149]. Arbutin can inhibit ROS-mediated signal transduction in melanocytes and prevent skin hyperpigmentation, like other dietary phenolic compounds [142]. In addition, the increased pool of intracellular thiol compounds may enhance pheomelanin synthesis and reduce eumelanin synthesis through the formation of DOPA-thiol conjugates [45,136,137]. The antioxidant action-based mechanism and the mechanism based on the TYR inhibitory action are not mutually exclusive and are assumed to work together for the inhibition of eumelanin synthesis (Figure 6).

There is still no consensus as to which arbutin or α-arbutin is better in terms of TYR inhibition, cell melanin synthesis inhibition, and skin lightening efficacy and safety. In addition, whether they inhibit cell melanin synthesis or show skin lightening action through the production of hydroquinone has not been completely resolved. Despite these controversies, the results of animal tests and clinical trials prove that arbutin and α-arbutin have the effect of alleviating skin pigmentation.

Arbutin and α-arbutin have the possibility to produce hydroquinone in the manufacturing process of raw materials, the manufacturing and storage process of cosmetic products, and the human use of the products. Whether they are chemically synthesized, extracted from plants, or produced by biological conversion processes, it is an important goal to achieve high purity in the final raw materials. The formulation is important to stabilize arbutin or α-arbutin in the final products. It should be remembered that exposure to microorganisms or UVR during storage and use of the product has the potential to generate hydroquinone [59,60]. Since dermatitis or allergic dermatitis has been observed in certain people using arbutin-containing cosmetic products [152,153], if any side effects are observed while using the product, one must discontinue use immediately and consult a doctor.

Substances and therapies that can synergize with arbutin are attracting attention [117,120,122]. Rather than using arbutin alone, a strategy for maximizing the skin lightening efficacy by combining it with other active ingredients with different mechanisms of action is preferred [124,125,126]. In future studies, it is hoped that more diverse active ingredients can be combined so that a highly synergistic increase in efficacy can be achieved. Techniques that aid in the transdermal absorption and efficient release of active ingredients are also needed. Nanoemulsions [110], nanoparticles [111,112], and microneedles [114] containing arbutin have been developed. Ultrasonic treatment with microbubbles is could promote percutaneous absorption [113]. Of course, a comprehensive treatment strategy that combines pharmacological treatment with laser treatment is expected to give added benefits [127].

## 10. Conclusions

In conclusion, the position of arbutin as a skin depigmenting agent is somewhere between the advantages of efficacy and the disadvantages of side effects. Therefore, knowledge of how cosmetic products containing arbutin are made, managed, and used is important. A hypothetical model for the depigmenting mechanism of arbutin involving antioxidant action as well as TYR inhibition is proposed. It is expected that more advanced depigmenting products will be developed based on accumulated information on arbutin and related substances. It is also expected that future research will examine whether arbutin can be applied to skin disorders other than hyperpigmentation.

## Figures and Tables

**Table 1:** Tyrosinase inhibitory effects of arbutin and α-arbutin.

Literature	Compounds	Tyrosinase Inhibitory Effects		Enzymes and Substrates Used
		Monophenolase Activity	Diphenolase Activity	
[73]	Hydroquinone	97.2% inhibition at 3 mM		Mushroom tyrosinase; 0.3 mM L-tyrosine
	Arbutin	82.0% inhibition at 3 mM		
	α-arbutin	72.8% inhibition at 3 mM		
[85]	α-arbutin		IC_50_ = 0.48 mM	B16 mouse tyrosinase; 3.3 mM L-DOPA
	Arbutin		IC_50_ = 4.8 mM	
	α-arbutin		No inhibition	Mushroom tyrosinase; 0.83 mM L-DOPA
	Arbutin		IC_50_ = 8.4 mM	
[87]	α-arbutin	IC_50_ = 8 mM	IC_50_ = 8.87 mM	Mushroom tyrosinase; 0.25 mM L-tyrosine plus 0.01 mM L-DOPA for monophenolase activity; 0.5 mM L-DOPA for diphenolase activity
	Arbutin	IC_50_ = 0.9 mM	IC_50_ = 0.7 mM	

**Table 2:** Tyrosinase inhibitory effects of arbutin and α-arbutin and their glucosides.

Literature	Compounds		Tyrosinase Inhibitory Effects		Enzymes and Substrates Used
	Name	Chemical Structure	Monophenolase Activity	Diphenolase Activity	
[89]	α-arbutin	3		IC_50_ = 2.1 mM	Human tyrosinase; 3.3 mM L-DOPA
	Arbutin	2		IC_50_ > 30 mM	
	4-Hydroxyphenyl β-maltoside	7		IC_50_ = 5.7 mM	
	4-Hydroxyphenyl β-maltotrioside	8		IC_50_ = 6.1 mM	
[90]	4-Hydroxyphenyl α-maltoside	11		IC_50_ = 4.9 mM	Human tyrosinase; 3.3 mM L-DOPA
	4-Hydroxyphenyl α-maltotrioside	12		IC_50_ = 13.9 mM	
[91]	Arbutin	2		K_i_ = 2.8 mM	Mushroom tyrosinase; 3.3 mM DOPA
	4-Hydroxyphenyl β-isomaltoside	9		K_i_ = 3.7 mM	
	4-Hydroxyphenyl β-isomaltotrioside	10		Not determined	
[92]	Arbutin	2	IC_50_ = 6 mM		Mushroom tyrosinase; 0.03% L-tyrosine
	β-D-Glucopyranosyl-(1→6)-arbutin	4	IC_50_ = 8 mM		
	β-D-Glucopyranosyl-(1→4)-arbutin	5	IC_50_ = 10 mM		
	β-D-Glucopyranosyl-(1→3)-arbutin	6	IC_50_ = 5 mM		
	α-D-Glucopyranosyl-(1→4)-arbutin	7	IC_50_ = 5 mM		
[93]	α-arbutin	3		IC_50_ = 2.1 mM	Human tyrosinase; 3 mM L-DOPA
	α-arbutin-α-glucoside	11		IC_50_ = 6.9 mM	
	α-arbutin-α-maltoside	12		IC_50_ = 15.6 mM	
	α-arbutin-α-maltotrioside	13		Not determined	

**Table 3:** Tyrosinase inhibitory effects of various esters of arbutin.

Literature	Compounds		Tyrosinase Inhibitory Effects		Enzymes and Substrates Used
	Name	Chemical Structure	Monophenolase Activity	Diphenolase Activity	
[95]	Arbutin	2	IC_50_ = 3 mM	IC_50_ = 40 mM	Mushroom tyrosinase; 1 mM catechol or 1 mM phenol
	Arbutin undecylenic acid ester	17	IC_50_ = 0.4 mM	IC_50_ = 0.4 mM	
[98]	Grevilloside M	19		No inhibition	Mushroom tyrosinase; 2.5 mM L-DOPA
	Robustaside D	20		No inhibition	
[96]	Arbutin	2	1.72% inhibition at 0.2 mM	Not detected	Mushroom tyrosinase; 2 mM L-tyrosine or 1 mM L-DOPA
	Arbutin propionate	15	0.86% inhibition at 0.2 mM	Not detected	
	Arbutin octylate	16	8.42% inhibition at 0.2 mM	Not detected	
	Arbutin undecenoate	17	15.64% inhibition at 0.2 mM	8.01% inhibition at 0.2 mM	
	Arbutin laurate	18	Not detected	Not detected	
[97]	Arbutin	2		IC_50_ = 29.4 mM	Silkworm hemolymph polyphenol oxidase; 14.4 mM L-DOPA
	Arbutin undecylenic acid ester	17		IC_50_ = 6.36 mM	

**Table 4:** Opinion of the scientific committee on consumer safety (SCCS) on the safety of the use of arbutin, α-arbutin, and deoxyarbutin in cosmetic products.

Literature	Compounds		Statements
	Name	Chemical Structure	
[103]	Arbutin	2	“The SCCS considers the use of β-arbutin to be safe for consumers in cosmetic products in a concentration up to 7% in face creams provided that the contamination of hydroquinone in the cosmetic formulations remain below 1 ppm.”
[104]	α-arbutin	3	“The SCCS considers the use of α-arbutin safe for consumers in cosmetic products in a concentration up to 2% in face creams and up to 0.5% in body lotions.”
[105]	Deoxyarbutin	22	“Therefore, the overall conclusion of the SCCS is that the use of deoxyarbutin up to 3% in face creams is not safe.”

**Table 5:** Effects of arbutin on the viability of different cells.

Literature	Cells	Effects of Arbutin on Cell Viability
[50,52]	Human melanocytes derived from neonatal Caucasian or Asian neonatal foreskins	Arbutin treatment at 0.01–1.0 mM for 3 d did not reduce cell viability whereas 5 mM treatment reduced cell viability by 26%.
[53]	Normal human melanocytes from foreskins of 18- to 40-year-old Japanese males	Cells grew well in the presence of 0.37 mM arbutin for 5 d, but 1.1 mM arbutin was cytotoxic and cells detached from the dish within 48 h.
[56]	BRUCE-4 embryonic stem cells of C57BL/6J mouse; Mouse bone marrow-derived stromal ST2 cells	Arbutin treatment for 24 h did not inhibit the proliferation of either cell at 1 mM.
[94]	Murine melanoma B16 cells	After 24 h of treatment, up to 3.6 mM arbutin had no significant effect on cell viability. After 48 h, up to 0.7 mM arbutin did not induce significant toxicity. After 72 h, 0.3–5.4 mM arbutin reduced cell viability by 24–45%. Arbutin at 5.4 mM induced apoptosis.
[143]	Normal human skin fibroblasts	Treatment with up to 1 mM arbutin for 24 h did not affect cell viability.
[151]	Human prostate carcinoma. The LNCaP cell line Human prostate carcinoma LNCaP cells	Treatment with 125–2000 μM for 24, 48, or 72 h did not significantly affect cell viability.Arbutin induced apoptosis at 1000 μM.
[147]	Fibroblast cell line from human newborn foreskins; LNCaP cells	Arbutin reduced the viability of these cells at doses above 1000 μM at 24 and 48 h post-exposure.
